# Within-individual changes in physical work demands associated with self-reported health and musculoskeletal symptoms: a cohort study among Dutch workers

**DOI:** 10.1007/s00420-023-02008-0

**Published:** 2023-09-25

**Authors:** Bart Cillekens, Emma van Eeghen, Karen M. Oude Hengel, Pieter Coenen

**Affiliations:** 1 Department of Public and Occupational Health, Amsterdam UMC, Location VU University Medical Center (VUmc), Van der Boechorststraat 7, 1081 BT Amsterdam, The Netherlands; 2 Amsterdam Public Health Research Institute, Societal Participation & Health, Amsterdam, The Netherlands; 3 Department of Work Health Technology, TNO, Sylviusweg 71, 2333 BE Leiden, The Netherlands

**Keywords:** Workload, Working conditions, Occupational physical activity, Health, Muscular system, Gender

## Abstract

**Purpose:**

This study aimed to investigate changes in physical work demands in association with self-rated health and musculoskeletal symptoms.

**Methods:**

Data from five waves over the period 2019–2021 of the Netherlands Working Conditions Survey COVID-19 were available for 7191 participants aged 19–64 years who worked (partly) on-site during at least two consecutive waves. Logistic generalized estimated equations (GEE) were used to estimate the odds ratios (OR) with 95% confidence interval (CI) for changes (increase or decrease compared to no change) in physical work demands between two waves and poor self-rated health and musculoskeletal symptoms in the following wave, adjusted for the health outcome at the first wave, age, educational level, working hours and hours worked from home.

**Results:**

In females, a statistically significant association was found between an increase in physical work demands compared to no change and musculoskeletal symptoms (OR 1.39, 95% CI 1.17–1.65). A decrease in physical work demands in females was not statistically significantly associated with musculoskeletal symptoms (OR 0.93, 95% CI 0.80–1.08). Similar trends were found for poor self-rated health, although non-statistically significant. For males, comparable but attenuated associations were found.

**Conclusion:**

While our study showed that increasing physical work demands are associated with adverse health (self-reported and musculoskeletal), it did not appear to benefit worker’s health to reduce work demands. Future research with multiple measurements in a shorter period and additionally using devices to measure physical work demands will be needed to confirm our study results.

**Supplementary Information:**

The online version contains supplementary material available at 10.1007/s00420-023-02008-0.

## Introduction

Workers exposed to physical work demands are more likely to report health problems than workers with mainly sedentary tasks (Mänty et al. 2022; Taimela et al. 2007). Physical work demands such as excessive repetition, heavy lifting and awkward postures are associated with an increased risk for musculoskeletal disorders (da Costa and Vieira 2010). For example, standing for long periods is associated with musculoskeletal disorders in feet, legs, hips and the lower back (Waters and Dick 2015). Also, heavy lifting and awkward postures, often apparent in physically demanding jobs, seem to play a major role in the development of work-related musculoskeletal disorders (da Costa and Vieira 2010). Higher physical work demands have also shown to be associated with other health problems including poor self-rated health (Proper et al. 2020), ischemic heart disease (Holtermann et al. 2009), atherosclerosis (Krause et al. 2007) and all-cause mortality in males (Coenen et al. 2018). Moreover, the negative consequences of high physical work demands resulted in a shortened working life, sickness absence and unemployment (Pedersen et al. 2020).

To date, most evidence on studies linking physical work demands with health outcomes are assessing the association of an exposure at baseline with outcomes after a follow up period (Cillekens et al. 2022). These associations between physical work demands and health outcomes are often based on a long-follow up period from years to even decades (Coenen et al. 2018; Holtermann et al. 2009). A baseline work demand as a predictor of an health outcome, assumes that physical work demands do not change during the follow up period. However, this is often not the case as people can change jobs and/or their work tasks can change in intensity during the course of a career (Åkerstedt et al. 2019), or across seasons (Grzywiński et al. 2022), workweeks or days (Sandlund et al. 2017).

Overall, most jobs have become less physically demanding in the last decades (Åkerstedt et al. 2019; Straker and Mathiassen 2009) through, for example, mechanization (Burdorf et al. 2007) or ergonomic innovations (Das et al. 2021). A repeated cross sectional study in the Netherlands showed that the mean physical work demand score (range 0–4) was 2.1 among a cohort 1993, and this decreased to 1.7 in 2003 and even to 1.4 in 2013 (van der Noordt et al. 2019). An example of a job changing in physical work demands was the Swedish forestry sector, where lumberjacks heavy working tasks (timber cutting and loading load) have mostly been replaced by forestry machines (Straker and Mathiassen 2009). Despite this, there is still a substantial proportion of the working population who are regularly exposed to high physical work demands (Venema 2020).

To get a better understanding of the impact of physical work demands, the role of changes in exposure level on worker’s health needs to be investigated. To date, only a few studies have investigated the association of changes in physical work demands on health and work participation (Badarin et al. 2022; Saastamoinen et al. 2014; van de Ven et al. 2022). Van de Ven et al. (2022) showed that in a sample of Dutch workers aged 45 and older, a decrease in physical work demands was associated with an improved work ability and self-rated health in the same year. They also showed that an increase in physical work demands was associated with a decreased work ability and adverse health (van de Ven et al. 2022). Similarly, a study among municipal employees in Helsinki aged 40–60 years found that reducing physical working conditions was followed by a lowered risk for sickness absence one year later, while an increase in this exposure was followed by an increased risk (Saastamoinen et al. 2014). Another study, in which over 300,000 workers were surveyed in Sweden with physically demanding jobs, showed that a change into lower physical work demands was associated with a reduced risk of all-cause and musculoskeletal disability pension for both genders (Badarin et al. 2022).

Based on the limited evidence on associations of changes in physical work demands and health, in the current study we will contribute to the literature in several ways. Firstly, we will examine the topic among a broad sample of workers, rather than focusing solely on older workers or those in specific sectors. Secondly, given the gender-specific associations between physical work demands and health outcomes (Badarin et al. 2022; Cillekens et al. 2022; Guettler 2023; Serna Arnau et al. 2023), we will also investigate potential gender differences in the association of changes in physical work demands on health. Thirdly, we will assess the association of changes in physical work demands on health within a relatively short time period (6 months) as we hypothesize that changes in physical work demands will result in acute changes in health. Therewith, the insights of our study will contribute to the development of future recommendations and interventions regarding how to prevent adverse health outcomes and potentially improve the health of workers in jobs with varying physical work demands. Therefore, the aim of our study is to examine the associations of within individual changes in physical work demands with self-rated health and musculoskeletal symptoms in Dutch workers, stratified by gender.

## Method

### Study design

This cohort study is embedded within ‘the Netherlands Working Conditions Survey COVID-19’ (NWCS-COVID-19), which is an ongoing follow up study of the annual NWCS 2019 (Hooftman et al.^2020^) that has extensively been described elsewhere (Oude Hengel et al. 2022). The cohort study was set out amongst Dutch workers between the age of 15 and 74 years and aimed to provide an insight into—among others—their working conditions and health over the period 2019–2021. For the NWCS-COVID-19 study, a group of 26,334 participants of the NWCS 2019 that gave permission to be approached again were asked to participate in the study in November 2019 (wave 1). The second measurement took place in July 2020 (wave 2); the third measurement took place in November 2020 (wave 3), the fourth in March 2021 (wave 4) and the fifth in November–December 2021 (wave 5). The study followed all recommendations with regard to ethical aspects, including an informed consent procedure after informing patients with an information letter. The TNO Internal Review Board approved the study and assessed the NWCS-COVID-19 cohort study as not being subject to the requirements of the Medical Research (Human Subjects) Act (ID number: 2019-061 for Wave 1; ID number: 2020-057 for Wave 2–4; ID number: 2021-101 for Wave 5). Other than wave 1, the rest of the measurements took place during the COVID-19 pandemic, in which the governmental measures to reduce the spread of the Sars-Cov-2 infections varied. During wave 2, the governmental measures were just relaxed (e.g., primary schools, day care and all occupational sectors were open). Wave 3 took place when the number of infections and hospitalizations were on a rise again, non-essential shops had restricted opening hours, and the restaurants, bars and entertainment industry were closed. During wave 4, on top of aforementioned restrictions, non-essential shops were only open upon appointment and there was an evening curfew. Also in this period, the vaccination campaign had started for essential workers and older workers. After a summer of relaxation of many regulations, more governmental measures (e.g., restaurants were closed) were taken again during wave 5.

### Study sample

**Fig. 1:**
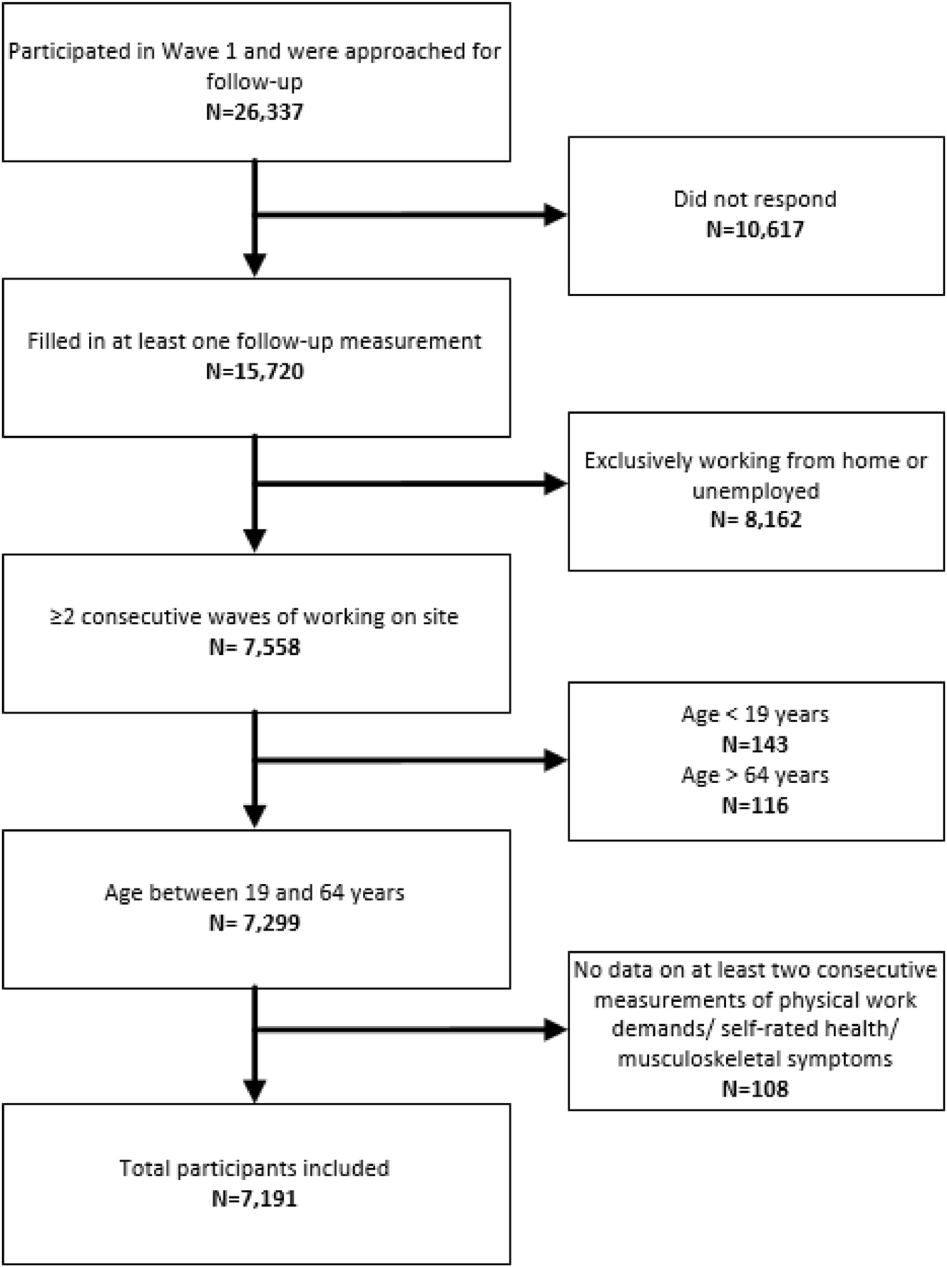
Flowchart of the included participants

Dutch workers aged 19–64 years at wave 1 were included in our study. To investigate the changes in physical work demands within participants, data on at least two consecutive measurements with data on exposure and outcomes were required (*n* = 15,720). As physical work demands were not measured in participants exclusively working from home or when temporarily unemployed, observations from such workers and their following observations were excluded (*n* = 8126). In total, the study consists of a study sample of 7191 workers with information from on average 3.2 waves (Fig. 1).

### Physical work demands

Physical work demands were measured using four different questions: ‘Does the work you perform require using a lot of force?’, ‘Do you use a tool, device or vehicle that causes vibrations or shaking?’, ‘Do you perform work in an uncomfortable position?’ and ‘Does your work require repeated movements?’ All questions could be answered with ‘yes, regularly’, ‘yes, sometimes’ and ‘no’. Answering option ‘no’ was scored a 1, ‘yes, sometimes’ a 2 and ‘yes regularly’ a 3. The mean score of the four items was calculated for each participant for each wave, resulting in a score ranging between 1 and 3. The Cronbach’s alpha for all questions measuring physical work demands was 0.78, indicating sufficient internal consistency across the four questions (Tavakol and Dennick 2011). At wave 1, participants were categorized into low (score: 1.00–1.74) and high physical work demands (score 1.75–3.00). For sensitivity analyses, the variable was categorized into: low (score 1.00–1.50), moderate (1.51–2.25) and high physical work demands (2.26–3.00).

For every two consecutive waves, for each participant, the change in physical work demands was calculated between the measurement (*T*_n_) and the following measurement (*T*_n+1_). The scores varied between − 2 (largest decrease in physical work demands possible; i.e., changing from 3 to 1) and 2 (largest increase in physical work demands possible; i.e., changing from 1 to 3). Due to a non-linear association that we observed between change in physical work demands and both self-rated health and musculoskeletal symptoms, the independent variable was treated as categorical variable with three categories: no change in physical work demands (change score between − 0.25 and 0.25), decrease in physical work demands (change scores < − 0.25) and increase in physical work demands (change score > 0.25). Scores < − 0.25 or > 0.25 represent at least one category change in any of the four questions on physical work demands. These arbitrary cut-off points were choses to make sure that all groups contained sufficient numbers of participants.

### Musculoskeletal symptoms

Musculoskeletal symptoms consisted of one question with multiple items. The main question was: ‘In the past 12 months (wave 1)/past three months (waves 2–5), have you suffered from pain, discomfort in your…?’ Participants could answer this question for the following body parts: (i) arms, elbows, shoulders, or neck, (ii) wrists or hands, (iii) back, and (iv) hips, legs, knees or feet. The five answering options were ‘No, never’, ‘Once in a while, but not for long’, ‘Once in a while, but long-lasting’, ‘Multiple times, but not for long’ and ‘Multiple times, long-lasting’. If the participant answered all items with ‘no, never’ or ‘once in a while, but not for long’, the participant was categorized as having no musculoskeletal symptoms. Others were categorized as having musculoskeletal symptoms.

### Self-rated health

Self-rated health was assessed using one question: ‘How is your health in general?’ Participants could answer the question with: ‘Excellent’, ‘Good’, ‘Fair’, ‘Poor’ and ‘Very poor’. Self-rated health is a strong predictor for morbidity and mortality (17). Self-rated health was dichotomized as good health (good/excellent) and poor (fair/poor/very poor) self-rated health.

### Covariates

Age, educational level, working hours and working hours from home were included as covariates. Age was assessed as a categorical variable with five categories (19–24, 25–34, 35–44, 45–54, and 55–64 years). Education level was assessed as the highest level achieved at wave 1, categorized as: low (intermediate secondary education or less), intermediate (higher general secondary education or intermediate vocational education) or high (higher vocational education or university). The number of working hours was a categorical variable (< 9, 9–16, 17–24, 25–32, 33–40, > 40 h per week). The percentage of hours worked from home was calculated based on the number of hours worked from home, divided by the total number of contracted hours per week and categorized into: < 20%, 20–39%, 40–79%, and ≥ 80%.

### Statistical analysis

Firstly, descriptive statistics were used to gain insight on participant characteristics at wave 1. Secondly, we conducted a logistic regression analysis to assess the association between physical work demands and both self-rated health and musculoskeletal symptoms at wave 1. As a third step, we performed a logistic generalized estimating equations (GEE) model to investigate the associations between a change in physical work demands (between *T*_n_ and *T*_n+1_) and poor self-rated health and musculoskeletal symptoms (*T*_n+1_). An exchangeable correlation structure was chosen after analyzing the observed correlation structures separately between waves. To test for multicollinearity between all included variables, a variance inflation factor (VIF) test was performed using a cut-off value of below 5.0 (Midi et al. 2010). Since all scores were found to be below 5.0, all covariates could be included in our analysis.

For both the logistic regressions and the GEE models, we firstly investigated the association between (change in) physical work demands and self-rated health and musculoskeletal symptoms in model 1. As the odds for poor self-rated health and having musculoskeletal symptoms measurements highly depended on previous musculoskeletal symptoms and self-rated health status, values of these outcomes at wave 1 were included in all GEE analyses. In model 2 we adjusted additionally for age and educational level. In model 3, we additionally adjusted for working hours and hours worked from home. All analyses were stratified by gender. Analyses were performed in STATA (version 14).

## Results

Slightly more than half of the study sample was female (56%) and half of them were highly educated (51%; Table 1). The majority of the workers were ≥ 45 years (45–54 years: 26% and 55–64 years: 32%). Female workers tended to report more often musculoskeletal symptoms (49%) than males (35%).

**Table 1:** Characteristics of the study sample at wave 1 (*n* = 7191)

	Total, *n* = 7191	Male, *n* = 3168	Female, *n* = 4023
Age, years (*n*, %)			
19–24	368 (5%)	138 (4%)	230 (6%)
25–34	1157 (16%)	433 (14%)	724 (18%)
35–44	1497 (21%)	638 (20%)	859 (21%)
45–54	1898 (26%)	863 (27%)	1035 (26%)
55–64	2271 (32%)	1096 (35%)	1175 (29%)
Highest attained education level (*n*, %)			
Low	687 (10%)	357 (11%)	330 (8%)
Intermediate	2787 (39%)	1376 (44%)	1411 (35%)
High	3688 (51%)	1417 (45%)	2271 (57%)
Working hours per week (mean, SD)	30.3 (10)	35.0 (9)	26.7 (9)
Low physical work demands	4922 (68%)	2056 (65%)	2866 (71)
High physical work demands^a^ (*n*, %)	2269 (32%)	1112 (35%)	1157 (29%)
Poor self-rated health^b^ (*n*, %)	1519 (21%)	665 (21%)	854 (21%)
Low physical work demands	878 (18%)	350 (17%)	528 (18%)
High^a^ physical work demands	641 (28%)	315 (28%)	326 (28%)
Good self-rated health (*n*, %)	5672 (79%)	2503 (79%)	3169 (79%)
Low physical work demands	4044 (71%)	1706 (68%)	2338 (74%)
High^a^ physical work demands	1628 (29%)	797 (32%)	831 (26%)
Musculoskeletal symptoms^c^ (*n*, %)	3109 (43%)	1123 (35%)	1986 (49%)
Low physical work demands	1933 (39%)	636 (31%)	1197 (45%)
High^a^ physical work demands	1176 (52%)	487 (44%)	689 (60%)
No musculoskeletal symptomsc (*n*, %)	4082 (57%)	2045 (65%)	2037 (51%)
Low physical work demands	2989 (73%)	1420 (69%)	1569 (77%)
High^a^ physical work demands	1093 (27%)	625 (31%)	468 (23%)

*n* number of participants, *SD* standard deviation

^a^High physical work demands means a score of ≥ 1.75

^b^Self rated health ‘fair/poor/very poor’

^c^When answered ‘yes, regularly’ at least once regarding using force, vibration/shaking, uncomfortable position or repetitive movements

Descriptive statistics of the changes of physical work demands between two consecutive waves are shown in Table 2. The physical work demands did not change between two waves for both females and males. For approximately 10% of the females and 11% of the males, a decrease in physical work demands was found between two waves, while this was 8% and 11%, respectively, for an increase in physical work demands between two waves. Online resource 1 presents an overview of industries in which females and males worked, stratified by change in physical work demands.

**Table 2:** Descriptive of changes in physical work demands between two consecutive waves

	No change in physical work demands	Decrease in physical work demands	Increase in physical work demands
Females			
Between wave 1 and 2	2530 (78%)	420 (13%)	290 (9%)
Between wave 2 and 3	1625 (85%)	141 (7%)	137 (7%)
Between wave 3 and 4	1446 (86%)	113 (7%)	117 (7%)
Between wave 4 and 5	1071 (86%)	94 (8%)	82 (7%)
Total observations	6672 (83%)	768 (10%)	626 (8%)
Males			
Between wave 1 and 2	1974 (76%)	343 (13%)	293 (11%)
Between wave 2 and 3	1078 (81%)	132 (10%)	121 (9%)
Between wave 3 and 4	922 (81%)	108 (9%)	113 (10%)
Between wave 4 and 5	720 (79%)	88 (10%)	105 (12%)
Total observations	4694 (78%)	671 (11%)	632 (11%)

*n* number of observations

### Associations of physical work demands and musculoskeletal symptoms and self-rated health at wave 1

At wave 1, the logistic regression models showed that for both female (OR 1.74, 95% CI 1.60–1.89 for model 3) and male workers (OR 2.04, 95% 1.84–2.25) high physical work demands were significantly associated with a higher odds of musculoskeletal symptoms compared to workers with low physical work demands (Table 3). Similar associations were found for poor self-rated health. Females (OR 1.75, 95% CI 1.57–1.94) and males (OR 1.65, 95% CI 1.47–1.87) with high physical work demands had a higher odds of poor self-rated health compared to workers with low physical work demands. Sensitivity analyses showed the same pattern when analyzing three groups of physical work demands using different cut-off values (Online Resource 2).

**Table 3:** Association between physical work demands and self-rated and musculoskeletal health at wave 1, stratified by gender

	Model 1^a^		Model 2^b*^		Model 3^c*^	
	OR	95% CI	OR	95% CI	OR	95% CI
Females						
Musculoskeletal symptoms (49%)						
Low physical work demands (N* = 2866, 71%)	Ref		Ref		Ref	
High physical work demands (N* = 1157, 29%)	1.78	1.55–1.79	1.69	1.56–1.83	1.74	1.60–1.89
Poor self-rated health (21%)						
Low physical work demands (N* = 2866, 71%)	Ref		Ref		Ref	
High physical work demands (N* = 1157, 29%)	1.84	1.67–2.02	1.75	1.58–1.93	1.75	1.57–1.94
Males						
Musculoskeletal symptoms (35%)						
Low physical work demands (N* = 2056, 65%)	Ref		Ref		Ref	
High physical work demands (N* = 1112, 35%)	1.90	1.74–2.07	1.98	1.80–2.18	2.04	1.84–2.25
Poor self-rated health (21%)						
Low physical work demands (N* = 2056, 65%)	Ref		Ref		Ref	
High physical work demands (N* = 1112, 35%)	1.74	1.57–1.93	1.57	1.40–1.76	1.65	1.47–1.87

*OR* odds ratio, *CI* confidence interval

*Number of participants in model 2 and 3 is lower, because of missing data on educational level (*N* = 22), working hours (*N* = 24) hours worked from home (*N* = 230)

^a^Unadjusted

^b^Adjusted for age and education level

^c^Adjusted for age, education level, working hours and hours worked from home

### Changes in physical work demands and musculoskeletal symptoms

Across all models, a statistically significant association was found between an increase in physical work demands between two waves and a higher risk of musculoskeletal symptoms compared to no change in physical work demands for females (OR 1.39, 95% CI 1.17–1.65 for model 3; Table 4). A decrease in physical work demands in females was not significantly associated with musculoskeletal symptoms compared to no change in physical work demands (OR 0.93, 95% CI 0.80–1.08). For males, similar trends were found, even though the odds ratios were more attenuated towards null and not statistically significant for both an increase and decrease in physical work demands and musculoskeletal symptoms [OR 1.15 (95% CI 0.95–1.39) and (OR 1.00, 95% CI 0.83–1.19) for model 3, for increasing and decreasing work demands, respectively].

**Table 4:** Associations between increases and decreases in physical work demands and musculoskeletal symptoms and poor self-rated health compared to no change in physical work demands among Dutch workers

		Model 1^a^			Model 2^b^			Model 3^c*^		
		OR	95% CI	Prevalence (%)^d^	OR	95% CI	Prevalence (%)^d^	OR	95% CI	Prevalenc (%)e^d^
	Physical work demands									
	**Females**									
Musculoskeletal symptoms	Decrease	0.94	0.81–1.08	40.7	0.93	0.80–1.08	40.7	0.93	0.80–1.08	40.8
	No change	Ref		42.1	Ref		42.3	Ref		42.3
	Increase	1.40	1.19–1.66	49.1	1.38	1.17–1.63	48.9	1.39	1.17–1.65	49.2
Poor self-rated health	Decrease	1.01	0.83–1.23	16.3	0.98	0.80–1.20	13.8	0.99	0.80–1.21	14.0
	No change	Ref		16.2	Ref		15.9	Ref		15.7
	Increase	1.26	1.01–1.56	18.7	1.23	0.98–1.53	17.1	1.24	0.99–1.56	17.1
	**Males**									
Musculoskeletal symptoms	Decrease	1.02	0.86–1.22	30.3	1.01	0.85–1.20	30.1	1.00	0.83–1.19	30.0
	No change	Ref		29.9	Ref		30.0	Ref		29.9
	Increase	1.19	0.99–1.43	33.1	1.16	0.97–1.40	32.7	1.15	0.95–1.39	32.4
Poor self-rated health	Decrease	0.84	0.68–1.05	14.1	0.81	0.65–1.00	13.8	0.84	0.67–1.05	14.0
	No change	Ref		15.8	Ref		15.9	Ref		15.7
	Increase	1.17	0.92–1.48	17.5	1.12	0.88–1.42	17.1	1.14	0.89–1.45	17.1

*OR* odds ratio, *CI* confidence interval

*Number of observations: Model 1: females (7997), males (5944). Model 2: females (7976), males (5915). Model 3: females (7803), males (5750)

^a^Adjusted for musculoskeletal symptoms or self-rated poor health in the first wave

^b^Additionally adjusted for age and education level

^c^Additionally adjusted for working hours and hours worked from home

^d^Margins 95% CI can be found in Online Resource 4

### Changes in physical work demands and self-rated health

An increase in physical work demands among females was statistically significantly associated with a higher odds of poor self-rated health in model 1 (OR 1.26, 95% CI 1.01–1.56), but not in model 2 and 3 (OR 1.24, 95% CI 0.99–1.56; model 3, Table 4). A decrease in physical work demands was not associated with poor self-rated health in any of the models (OR 0.99, 95% CI 0.80–1.21 for model 3). For males, no significant associations were found for either an increase or decrease in physical work demands (OR 1.14, 95% CI 0.89–1.45 and OR: 0.84, 95% CI 0.67–1.05 for model 3, respectively).

## Discussion

For females, an increase in physical work demands was followed by a higher odds of musculoskeletal symptoms and poor self-rated health, even though the latter was not statistically significant. A decrease in physical work demands was not associated with either musculoskeletal symptoms or poor self-rated health among females. While similar patterns for all outcomes were found for males, none of the associations were statistically significant.

In line with previous studies investigating the association between an increase in physical work demands and self-rated health (van de Ven et al. 2022) and sickness absence (Saastamoinen et al. 2014), we found associations between an increase in physical work demands and poor health and musculoskeletal symptoms, especially among female. However, our study did not find a beneficial health effect of a decrease in physical work demands, which is contrary to the findings of these two studies and to a study among workers with heavy physical workloads conducted in Sweden (Badarin et al. 2022). It is important to note that there are differences in follow-up period, study sample, and methods between our study and previous studies. Regarding the follow-up period, our study investigated changes over a period of approximately six months, while the other studies used longer follow-up periods between one and three years. A shorter follow-up time in studies can be considered a strength because it allows for a closer temporal association between the exposure and outcome (Schram et al. 2020), and reduces the likelihood that other factors, such as changes in lifestyle or development of other health conditions, may have influenced the association (Andersen 2004). On the other hand, a longer interval between measurements can provide a more comprehensive picture of the impact of changes in physical work demands on health outcomes, as it allows for a longer period of time for any changes to occur and for potential long-term effects to become apparent.

Regarding the study sample, our study consisted of a sample from a general working population from all ages while previous studies consisted of older workers starting from 40 years of age (Badarin et al. 2022; Saastamoinen et al. 2014; van de Ven et al. 2022). Older workers might have a higher chance of developing adverse health outcomes (Rodgers et al. 2019; Safiri et al. 2021). In addition, while aging workers might gradually decline in their cardiovascular and musculoskeletal fitness, the physical work demands might remain the same, which may lead to an imbalance between the two (Ilmarinen et al. 1997; Kenny et al. 2008; Suorsa et al. 2022). This could lead to more adverse health events among older workers. However, it is also possible that the imbalance may have forced workers into less physically demanding work or exiting the labor market, known as the healthy worker effect (Guettler 2023; Hartvigsen et al. 2001).

One possible explanation for our finding that an increase in physical work demands was associated with negative health outcomes, but a decrease in physical work demands was not associated with positive health outcomes, could be related to the chronic and long-lasting nature of musculoskeletal symptoms (El-Tallawy et al. 2021). Participants who already had musculoskeletal symptoms and changed to a lower physically demanding job may still experience chronic symptoms, despite the decrease in physical work demands. Additionally, due to the short follow-up interval of our study, it is possible that an increase in demands had an acute negative effect on health, while a longer follow-up period may be needed to capture more gradual changes in health following a decrease in physical work demands.

We showed stronger associations between changes in physical work demands and health outcomes among females than males. Although the reasons for the differing associations between genders are not fully understood yet, some suggestions have been made. Firstly, in our sample females and males worked in different industries with females more often working in health care and males more often in manufacturing. As a result, physical work demands may differ between genders, with males having a higher proportion of tasks involving heavy lifting and handling of loads; females more often lift and move people (Serna Arnau et al. 2023). Even individuals with the same job title may have different physical work demands based on their gender (Guettler 2023). Secondly, females are more likely than males to develop musculoskeletal symptoms when high physical work demands are performed (Campos-Serna et al. 2013; Nordander et al. 2008). In our study, the prevalence of musculoskeletal symptoms at baseline was consistently higher in females (49%) than in males (35%). It is possible that physiological gender differences could account for the observed differences in effect sizes. For instance, males and females differ in physical capacity, muscle mass, and hormone status, among other factors (Allesøe et al. 2023; Guettler 2023). Thirdly, initial musculoskeletal symptoms may not solely be caused by changes in physical work demands, but also by other factors such as psychosocial, organizational, and individual factors that changed during the studied period and that could differ between genders.

### Strengths and limitations

Several strengths and limitations need to be taken into account. One of the strengths is that our study is among one of the firsts to investigate changes in physical work demands and their associations with health outcomes. Most literature on the health consequences of physical work demands is based on exclusively single measurements of the exposure (physical work demands) at baseline, while our study included changes in exposure on outcome at different time points, to identify within-individual changes in physical work demands over time and their association with health. Some limitations need to be considered as well. Firstly, the results rely on self-reported data, which is considered less valid and reliable than objective measurements. Secondly, the dichotomization of work demands and health outcomes may have resulted in a loss of information as a result of which we may not have detected all changes in exposure and health. Participants could have (small) within-individual improvements or deteriorations (e.g., moving from poor to fair health), which may not always have resulted in an improvement/deterioration in the categories that we used (Smith and Beaton 2008). This could possibly have led to attenuation of our findings, causing the null-findings (Grandjean et al. 2004). Thirdly, the wording of the question regarding musculoskeletal symptoms was changed during the study period. Where in the first wave the participants were asked if they had experienced pain or “in the past 12 months” this changed to “in the past three months” in the following waves. Fourth, residual and uncontrolled confounding of relevant factors, such as body mass index, leisure-time physical activity, or smoking, could not be ruled out as these were not measured in our study. Four out of five measurements took place during the COVID-19 pandemic. Within this time period, the governmental measures to stop the spread of the virus differed between measurements but also the working circumstances within this whole time period in various sectors differed from other years. For example, the number of homeworkers, and the number of hours working from home increased significantly during this period (Wiezer et al. 2022). The pandemic might also have influenced the health status, especially the self-rated health of the participants. This also may have confounded our associations, for example, by factors such as psychological and emotional stress (Adanaqué-Bravo et al. 2022), financial concerns and/or an infection with COVID-19.

### Implications

Insights found in this study can provide a starting point in developing future recommendations on how to prevent adverse health outcomes in workers. Especially if people increase their physical work demands, it seems important to provide tools or training on helping them to prevent adverse health outcomes. By taking a proactive approach to preventing adverse health outcomes in workers, employers can promote the health and well-being of their employees while also potentially reducing costs associated with absenteeism, disability, and healthcare utilization.

Since this study is one of the firsts to investigate the association between changes in physical work conditions and health, more research is needed to better understand the topic of changes in working conditions and health. To improve the assessment of physical work conditions, future studies could use more detailed measures such as device-based measurements that provide more detailed information on the physical demands of the job over the working day. Moreover, as daily lives of workers with physically demanding work vary day-by-day, it is important to gain insights into this variation in health over time, but also what working conditions predict such variability. Moreover, due to the current mobile technologies, ecological momentary assessments provide the opportunity to examine how exposures and outcomes vary and co-vary within-persons, over time, and across contexts (Asare et al. 2023).

## Conclusion

In conclusion, female workers increasing their physical work demands within a time period of six months were more likely to develop musculoskeletal symptoms and report poorer self-rated health than those who did not change their work demands. Although similar patterns were observed among males, the associations were not statistically significant. In both males and females, a decrease in physical work demands was not associated with a risk of adverse health outcomes. Future research should focus on identifying effective strategies to prevent adverse health outcomes among workers performing physically demanding work. Additionally, further studies with longer follow-up periods, multiple measurements within shorter intervals, and improved measurement methodologies for both physical work demands and health outcomes are needed to better understand the associations between changes in physical work demands and health outcomes.

### Supplementary Information

Below is the link to the electronic supplementary material.

Supplementary file1 (DOCX 60 KB)

## Data Availability

Data are stored at TNO, Unit Healthy Living in the Netherlands. Data are available upon reasonable request by the third author.
