# Supplementary material for: Within-individual changes in physical work demands associated with self-reported health and musculoskeletal symptoms: a cohort study among Dutch workers
Source: Int Arch Occup Environ Health. 2023 Sep 25;96(9):1301–11. doi: 10.1007/s00420-023-02008-0 (PMC10560189; doi:10.1007/s00420-023-02008-0)
Supplement: Supplementary file 1 — Supplementary file1 (DOCX 60 KB) [file 420_2023_2008_MOESM1_ESM.docx]

**Online Resource 1:** **Table S1:** Industries in which participants worked, stratified by change in physical work demands and by gender (displaying the top 5 sectors in each classification).

|  | **Change in physical work demands** | **Sector** | **N* (%)** |
| --- | --- | --- | --- |
| **Females** | No change in physical work demands | Education | 2,481 (37) |
|  |  | Healthcare | 2,233 (34) |
|  |  | Commerce | 541 (8) |
|  |  | Business | 499 (8) |
|  |  | Manufacturing | 253(4) |
|  | Decrease in physical work | Healthcare | 326 (42) |
|  |  | Education | 185 (24) |
|  |  | Commerce | 94 (12) |
|  |  | Business | 36 (5) |
|  |  | Hospitality | 32 (4) |
|  | Increase in physical work demands | Healthcare | 254 (41) |
|  |  | Education | 150 (24) |
|  |  | Commerce | 71 (11) |
|  |  | Business | 48 (8) |
|  |  | Manufacturing | 27 (4) |
| **Males** | No change in physical work demands | Education | 930 (20) |
|  |  | Manufacturing | 919 (20) |
|  |  | Commerce | 628 (13) |
|  |  | Business | 557 (12) |
|  |  | Healthcare | 411 (9) |
|  | Decrease in physical work | Manufacturing | 172 (26) |
|  |  | Commerce | 127 (19) |
|  |  | Education | 69 (10) |
|  |  | Business | 66 (10) |
|  |  | Healthcare | 56 (8) |
|  | Increase in physical work demands | Manufacturing | 159 (25) |
|  |  | Commerce | 118 (19) |
|  |  | Education | 80 (13) |
|  |  | Business | 64 (10) |
|  |  | Transportation | 64 (10) |
| N* = number of observations | | | |

**Online Resource 2**

| **Table S2:** Sensitivity analysis. showing the association between physically demanding work (three groups) and self-rated and musculoskeletal health at wave 1 | | | | |
| --- | --- | --- | --- | --- |
|  |  |  | Model 3^b^ | |
|  |  | N (%) | OR | 95% CI |
|  | **Females** |  |  | |
| Poor self-rated health | Low physical work demands^a^ | 2,716 (70) | Ref |  |
|  | Moderate physical work demands | 875 (23) | 1.58 | 1.31-1.91 |
|  | High physical work demands | 264 (7) | 2.29 | 1.73-3.04 |
| Musculoskeletal  symptoms | Low physical work demands | 2,716 (70) | Ref |  |
|  | Moderate physical work demands | 875 (23) | 1.79 | 1.52-2.11 |
|  | High physical work demands | 264 (7) | 2.18 | 1.67-2.85 |
|  | **Males** |  |  | |
| Poor self-rated health | Low physical work demands^a^ | 1,970 (64) | Ref |  |
|  | Moderate physical work demands | 713 (23) | 1.67 | 1.34-2.09 |
|  | High physical work demands | 385 (13) | 2.25 | 1.73-2.94 |
| Musculoskeletal  symptoms | Low physical work demands | 1,970 (64) | Ref |  |
|  | Moderate physical work demands | 713 (23) | 1.44 | 1.19-1.75 |
|  | High physical work demands | 385 (13) | 2.92 | 2.30-3.70 |
| OR=Odds Ratio; CI=Confidence interval.  ^a^low (between 1.00-1.50). moderate (between 1.51-2.25). high physical work demands (between 2.26-3.00)  ^b^adjusted for age, education level, working hours and hours worked from home. | | | | |

| **Online Resource 3: Corrected margins of self-rated health and musculoskeletal health.** | | | | | | | |
| --- | --- | --- | --- | --- | --- | --- | --- |
|  |  | Model 1^a^ | | Model 2^b^ | | Model 3^c*^ | |
|  |  | Margin | 95% CI | Margin | 95% CI | Margin | 95% CI |
|  | **Females** |  |  |  |  |  |  |
| Musculoskeletal  Symptoms | Decrease in physical work | 40.7 | 37.7-43.7 | 40.7 | 37.8-43.7 | 40.8 | 37.8-43.8 |
|  | No change in physical work demands | 42.1 | 40.1-43.4 | 42.3 | 41.0-43.6 | 42.3 | 41.0-43.7 |
|  | Increase in physical work demands | 49.1 | 45.7-52.6 | 48.9 | 45.5-52.3 | 49.2 | 45.7-52.6 |
| Poor self-rated health | Decrease in physical work demands | 16.3 | 14.2-18.3 | 13.8 | 11.7-15.8 | 14.0 | 11.9-16.1 |
|  | No change in physical work demands | 16.2 | 15.2-17.1 | 15.9 | 14.8-17.0 | 15.7 | 14.6-16.8 |
|  | Increase in physical work demands | 18.7 | 16.2-21.3 | 17.1 | 14.6-19.7 | 17.1 | 14.5-19.7 |
|  | **Males** |  |  |  |  |  |  |
| Musculoskeletal  symptoms | Decrease in physical work demands | 30.3 | 27.4-33.2 | 30.1 | 27.2-33.0 | 30.0 | 27.0-32.9 |
|  | No change in physical work demands | 29.9 | 28.5-31.3 | 30.0 | 28.6-31.4 | 29.9 | 28.6-31.4 |
|  | Increase in physical work demands | 33.1 | 29.9-36.3 | 32.7 | 29.5-35.9 | 32.4 | 29.2-35.7 |
| Poor self-rated health | Decrease in physical work demands | 14.1 | 12.0-16.2 | 13.8 | 11.7-15.8 | 14.0 | 11.9-16.1 |
|  | No change in physical work demands | 15.8 | 14.7-16.9 | 15.9 | 14.8-17.0 | 15.7 | 14.6-16.8 |
|  | Increase in physical work demands | 17.5 | 15.0-20.1 | 17.1 | 14.6-19.7 | 17.1 | 14.6-19.7 |
| ^a^Adjusted for musculoskeletal symptoms or self-rated poor health in the first wave. ^b^  additionally adjusted for age and education level. ^c^ additionally adjusted for working hours and hours worked from home. | | | | | | | |
